# Radiation therapy for recurrent extrahepatic bile duct cancer

**DOI:** 10.1371/journal.pone.0253285

**Published:** 2021-06-16

**Authors:** Minji Koh, Jin-hong Park, Changhoon Yoo, Sang Min Yoon, Jinhong Jung, Baek-Yeol Ryoo, Heung-Moon Chang, Kyu-pyo Kim, Jae Ho Jeong, Jong Hoon Kim

**Affiliations:** 1 Department of Radiation Oncology, Asan Medical Center, University of Ulsan College of Medicine, Seoul, Republic of Korea; 2 Department of Oncology, Asan Medical Center, University of Ulsan College of Medicine, Seoul, Republic of Korea; University of Wisconsin, UNITED STATES

## Abstract

**Purpose:**

More than half of patients with bile duct cancer (BDC) develop recurrence even after curative resection. Recurrent BDC has a poor prognosis, and no optimal treatment modality has been established. We therefore analyzed our experience on the survival outcomes of radiation therapy (RT) for recurrent extrahepatic bile duct cancer (EHBDC).

**Patients and methods:**

We retrospectively analyzed the records of patients with recurrent EHBDC who underwent concurrent chemoradiation therapy (CCRT) or RT alone at our institution between January 2001 and June 2015. Freedom from locoregional progression (FFLP), progression-free survival (PFS), and overall survival (OS) were assessed, and univariate and multivariate analyses were performed to identify the prognostic factors.

**Results:**

A total of 76 patients were included in the analysis. The median OS was 16 months and the rates of 2-year FFLP, PFS, and OS were 61%, 25%, and 33%, respectively. Among the evaluable patients, the first site of failure was the locoregional area in 16 patients, distant metastasis in 27, and both sites in 8. On univariate analysis, disease-free interval (*p* = 0.012) and concurrent chemotherapy (*p* = 0.040) were found as significant prognostic factors for OS. One patient with CCRT developed a grade 3 hematologic toxicity, and two patients experienced late grade 3 toxicities including duodenal ulcer bleeding and obstruction.

**Conclusions:**

RT for recurrent EHBDC showed favorable survival and local control with limited treatment-related toxicities. Considering that the most common pattern of failure was distant metastasis, further studies on the optimal scheme of chemotherapy and RT are warranted.

## Introduction

Bile duct cancer (BDC) is a rare malignancy with a poor prognosis. According to the site of the origin, BDC is classified into intrahepatic, perihilar, and distal BDC. Extrahepatic bile duct cancer (EHBDC) includes both perihilar and distal tumors. Surgical resection is the only curative treatment option for locoregional EHBDC, but surgical candidates with curative aim are generally limited [1]. Although EHBDC has a better prognosis than intrahepatic BDC (IHBDC) after curative resection, 40% to 76% of the patients still experience recurrence [2–4].

Importantly, isolated locoregional diseases account for nearly 60% of the first site of failure following curative surgery for perihilar BDC [5]. For locoregional recurrence of EHBDC, which could be potentially cured with complete local control (LC), an optimal treatment option is yet to be established. Studies on surgical resection for recurrent BDC reported a median survival duration of 19 months and a 3-year survival rate of 32%, which are significantly better than those in untreated patients [6, 7]. Unfortunately, not all patients can be indicated for salvage operation because of their general condition or the site of recurrence. In this aspect, radiation therapy (RT) can be a feasible treatment option for those unsuitable for surgical resection.

The clinical outcome of RT for recurrent BDC has been rarely reported, and the existing reports have small sample sizes or are case series on the results of RT for recurrent BDC [8–12]. We therefore evaluated the outcomes of RT in patients with locoregional recurrence of EHBDC treated at our institution.

## Materials and methods

### Patients

This retrospective study was reviewed by the institutional review board of Asan Medical Center (Seoul, Korea). The patients’ medical records were accessed between February 2017 and December 2018, and all data were fully anonymized before access. A total of 93 patients received salvage radiotherapy with a definitive aim for locoregional recurrence of EHBDC at our center between January 2001 and June 2015. In order to obtain a homogeneous group of patients, we excluded patients who were treated with stereotactic body radiotherapy or hypofractionated RT (n = 8) and those with incomplete RT before a total dose of 40 Gy due to disease progression or cholangitis (n = 9). All patients underwent curative resection at the time of initial diagnosis and had no evidence of disease before recurrence.

### Patient evaluation

For variables such as tumor location, pathologic T stage, pathologic N stage, and resection margin (RM), we used the data obtained at the initial diagnosis. For other patient characteristics, we used the data obtained at the time of the diagnosis of recurrence. Most of the cases of local recurrence were diagnosed using computed tomography (CT); five patients were assessed with magnetic resonance imaging (MRI), and six patients were evaluated with both CT and MRI. Positron emission tomography-computed tomography (PET-CT) was conducted in 54 patients. The diagnosis of recurrence and evaluation of the site of recurrence was mainly made clinically by the radiologists at our institution. Histological confirmation of recurrence was made by endoscopic biopsy in two patients and by percutaneous core needle biopsy in one patient. One patient underwent bile duct resection followed by RT to the porta hepatis lymph node. Carbohydrate antigen 19–9 (CA 19–9) level at the time of recurrence was higher than the upper normal limit in 38 patients (50%).

### Treatment

All patients were treated with 3-dimensional conformal radiotherapy (3D-CRT) and underwent CT simulation for treatment planning. The gross target volume (GTV) was the recurrent mass observed in CT images. The clinical target volume (CTV) was delineated by including regional lymph nodes or the RM area, in cases that were determined to be necessary. The planning target volume (PTV) was given with a nonuniform 1–2 cm margin considering setup uncertainty and breathing motion. All patients were treated with a total dose of 40–64 Gy (median, 54 Gy) with a daily dose of 1.8–3.0 Gy. The RT dose was scaled with biologically effective dose (BED) for the analysis. The total BED was 50.0–76.8 Gy (median, 64.8 Gy).

Concurrent chemotherapy was combined in 61 patients. Capecitabine (1250 mg/m^2^/day) was used in 47 patients, and eight patients received 5-fluorouracil (375 mg/m^2^/day) and leucovorin (20 mg/m^2^/day) by bolus intravenous injection in the first and fifth week of RT. Tegafur/uracil (300 mg/m^2^/day) with leucovorin was administered in six patients. Additional chemotherapy after RT was given to 25 patients with various regimens including gemcitabine plus cisplatin (n = 7), capecitabine plus cisplatin (n = 7), and capecitabine alone (n = 4). A total of 16 patients received chemotherapy prior to RT: adjuvant chemotherapy following initial curative resection (n = 12), chemotherapy for the recurrence before administration of RT (n = 6), and both following initial curative resection and before RT for recurrent disease (n = 2).

### Follow-up and statistical analysis

Regular follow-up evaluations including abdominopelvic CT scan, chest radiography, and laboratory tests were performed at intervals of 2 to 3 months after RT. Locoregional progression was defined as progression of the treated disease by using the Response Evaluation Criteria In Solid Tumors (RECIST) criteria (version 1.1) or newly developed lesion around the primary tumor location and regional nodal area. Distant metastasis was defined as recurrence in a systemic organ, the peritoneum, or a distant lymph node. The treatment-related toxicity was assessed using Common Terminology Criteria for Adverse Events (version 4.0).

Kaplan-Meier method was used to evaluate the freedom from locoregional progression (FFLP), progression-free survival (PFS), and overall survival (OS). All clinical endpoints were measured from the start date of RT. FFLP was calculated to the date of locoregional progression. OS was estimated until death and PFS was estimated until any site of tumor progression or death. Statistical significance was evaluated by the log-rank test. Cox regression method was used to estimate the effect of the selected prognostic factors. Variables with *p*-values less than 0.05 were regarded as statistically significant. IBM^®^ SPSS Statistics^®^ for Windows, version 22.0 (IBM Corp., Armonk, NY, USA) was used for statistical analysis.

## Results

A total of 93 patients received salvage radiotherapy as a definitive therapy for locoregional recurrence of EHBDC at our center during the study period. After applying the exclusion criteria, a total of 76 patients were included in the analysis and their characteristics at the time of recurrence or initial diagnosis are described in Table 1. Of the patients, 74% (n = 56) were males and the median age was 65 years (range, 22–78). The primary tumor site at the initial diagnosis was distal bile duct in 43 (57%) patients and perihilar bile duct in 33 (43%) patients. Twenty-two (29%) patients had pathologically proven nodal disease and 23 (30%) had positive RM at initial surgical resection. The patients were followed for a median of 13 months (range, 2–119). At the time of analysis, eight patients were alive without disease progression except for one patient with newly developed retroperitoneal lymph node metastases.

**Table 1:** Patient characteristics.

Characteristic^a^		No. (%)
Age (years)	Median (range)	65 (22–78)
Sex	Male	56 (74)
	Female	20 (26)
PS (ECOG)	0	17 (22)
	1	50 (66)
	2	9 (12)
Initial disease location	Perihilar	33 (43)
	Distal	43 (57)
Pathologic T stage	T1	11 (15)
	T2	33 (43)
	T3	32 (42)
Pathologic N stage	N0	53 (70)
	N1	22 (29)
	N/A	1 (1)
RM status	Negative	52 (69)
	Positive	23 (30)
	N/A	1 (1)
Disease-free interval	≤ 1 year	39 (51)
	> 1 year	37 (49)
Recurrence site^b^	Resection site	28 (37)
	Lymph node	41 (54)
	Anastomotic site	14 (18)
CA 19–9 (U/mL)	≤ 37	36 (47)
	> 37	38 (50)
	N/A	2 (3)
RT dose (BED)	≥ 59 Gy	68 (89)
	< 59 Gy	8 (11)
Concurrent chemotherapy	Yes	61 (80)
	No	15 (20)
Additional chemotherapy after RT	Yes	24 (32)
	No	52 (68)

PS, performance status; ECOG, Eastern Cooperative Oncology Group; N/A, Not assessed; RM, resection margin; CA 19–9, Carbohydrate antigen 19–9; RT, radiation therapy; BED, biologically effective dose.

^a^The characteristics were evaluated at the time of diagnosis of recurrence, except the initial disease treatment information including initial disease location, pathologic T stage, pathologic N stage, and RM status.

^b^Six patients showed both resection site and lymph node recurrence, and one patient showed both lymph node and anastomotic site recurrence.

**Fig 1:**
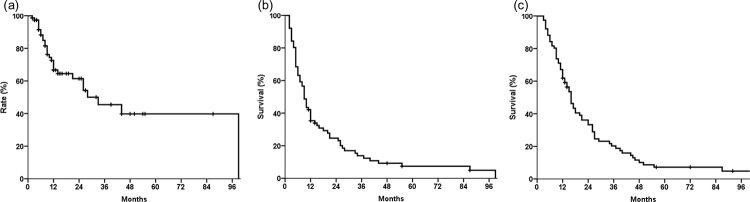
Survival and recurrence of the study patients. (a) Freedom from locoregional progression (FFLP). (b) Progression-free survival (PFS). (c) Overall survival (OS).

Fig 1 shows the survival curve of the patients. The 1-year OS rate was 62% and the median OS was 16 months (95% confidence interval (CI), 13–19 months). The 1-year rates of PFS (median, 9 months; 95% CI, 7–11 months) and FFLP (median, 33 months; 95% CI, 15–51 months) were 35% and 67%, respectively. The 2-year rates of OS, PFS, and FFLP were 33%, 25%, and 61%, respectively.

**Fig 2:**
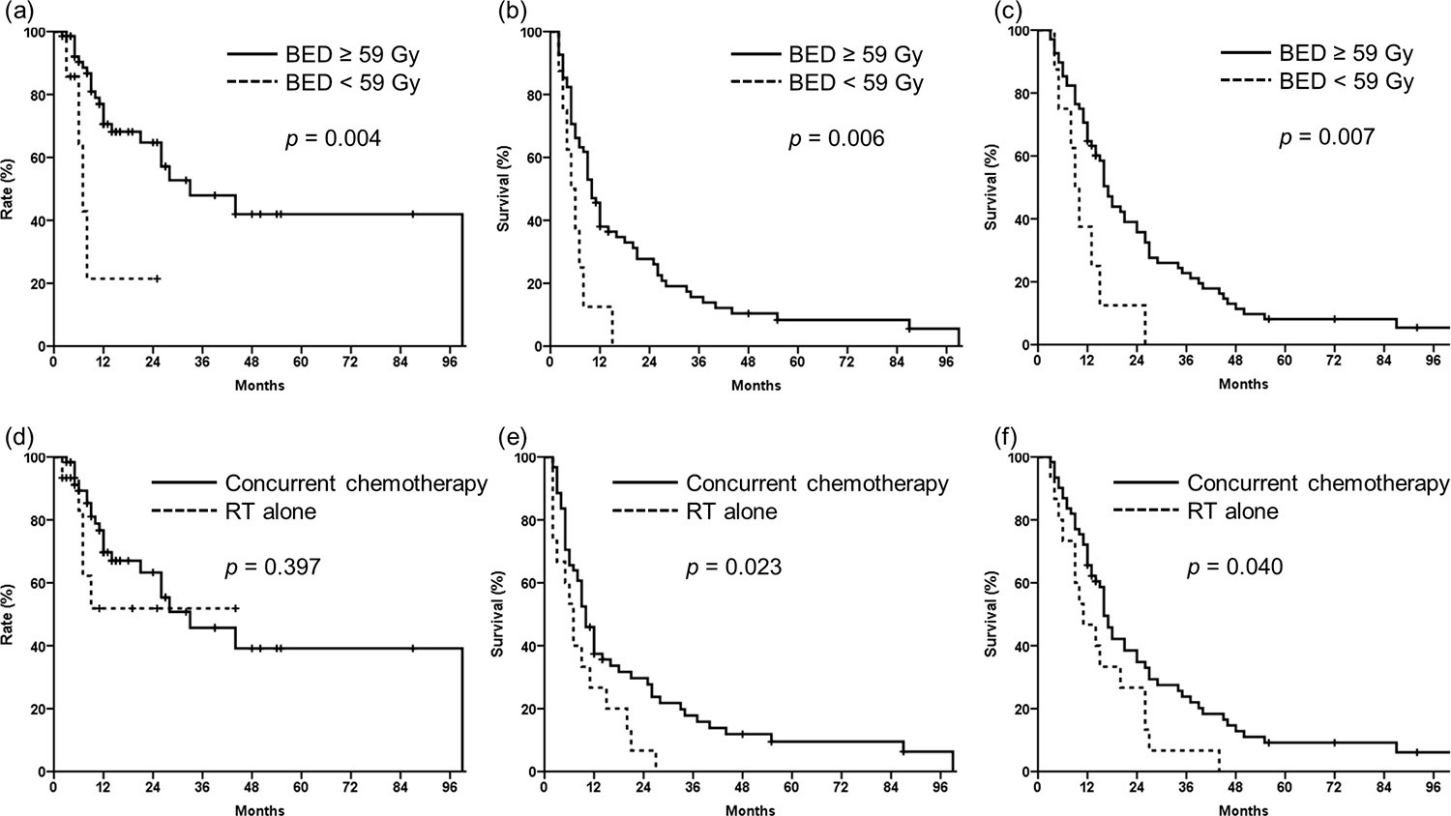
Survival and recurrence of the study patients. (a) Freedom from locoregional progression (FFLP), (b) Progression-free survival (PFS), (c) Overall survival (OS) according to biologically effective dose, and (d) FFLP, (e) PFS, (f) OS according to concurrent chemotherapy.

In univariate analysis, BED of 59 Gy or more was found to be an indicator for better OS, PFS, and FFLP (p = 0.007, 0.006, and 0.004, respectively; Table 2 and Fig 2A–2C). Patients with disease-free intervals (DFIs) of more than 1 year showed a significantly better PFS (43% vs. 28%, *p* = 0.042) and OS (70% vs. 54%, *p* = 0.012) than did those with shorter DFIs. Patients with increased levels of CA 19–9 at recurrence showed a significantly lower FFLP (75% vs. 53%, p = 0.025), and tended to have a lower PFS (41% vs. 26%, *p* = 0.073) and OS (72% vs. 50%, *p* = 0.056). Patients with concurrent chemotherapy during RT showed higher PFS (37% vs. 27%, *p* = 0.023, Fig 2E) and OS (66% vs. 47%, *p* = 0.040, Fig 2F) and additional chemotherapy tended to be associated with improved PFS (58% vs. 25%, *p* = 0.056). On multivariate analysis, DFI remained a significantly independent prognostic factor for OS (hazard ratio, 1.765; 95% confidence interval, 1.075–2.897, *p* = 0.025).

**Table 2:** Prognostic factors for survival (univariate analysis).

Prognostic factor^a^		No.	1-year FFLP (%)	*p*-value	1-year PFS (%)	*p*-value	1-year OS (%)	*p*-value
Sex	Male	56	69	0.612	35	0.317	63	0.585
	Female	20	59		35		60	
Age (years)	< 60	21	62	0.333	42	0.884	71	0.719
	≥ 60	55	69		33		58	
PS (ECOG)	0–1	67	70	0.380	36	0.626	63	0.825
	2	9	47		33		56	
Initial disease location	Perihilar	33	68	0.405	39	0.348	70	0.072
	Distal	43	66		33		56	
Pathologic T stage	T1	11	70	0.297	55	0.079	73	0.054
	T2-3	65	66		32		60	
Pathologic N stage	N0	53	71	0.586	39	0.335	66	0.073
	N1	22	64		27		55	
RM status	Negative	52	67	0.561	35	0.860	62	0.738
	Positive	23	67		39		65	
Disease-free interval	> 1 year	37	74	0.349	43	0.042	70	0.012
	≤ 1 year	39	59		28		54	
CA 19–9 (U/mL)	≤ 37	36	75	0.025	41	0.073	72	0.056
	> 37	38	53		26		50	
RT dose (BED)	≥ 59 Gy	68	71	0.004	38	0.006	65	0.007
	< 59 Gy	8	21		13		38	
Concurrent chemotherapy	Yes	61	70	0.397	37	0.023	66	0.040
	No	15	52		27		47	
Additional chemotherapy after RT	Yes	24	81	0.450	58	0.056	75	0.180
	No	52	60		25		56	

FFLP, freedom from locoregional progression; PFS, progression-free survival; OS, overall survival; PS, performance status; ECOG, Eastern Cooperative Oncology Group; RM, resection margin; CA 19–9, Carbohydrate antigen 19–9; RT, radiation therapy; BED, biologically effective dose.

^a^The characteristics were evaluated at the time of diagnosis of recurrence, except the initial disease treatment information including initial disease location, pathologic T stage, pathologic N stage, and RM status.

**Fig 3:**
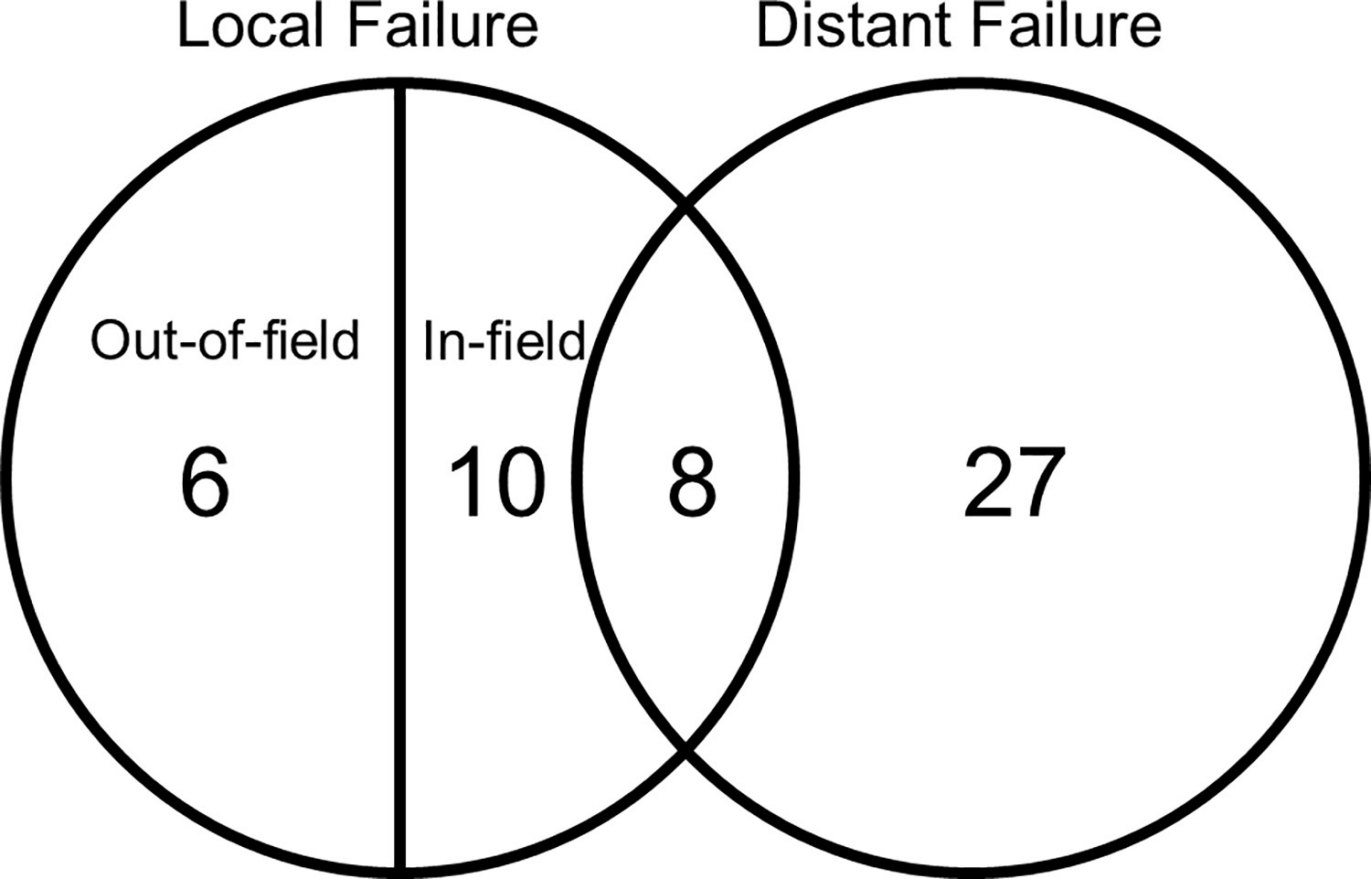
Pattern of failure.

During follow-up, 51 patients were evaluated for the site of disease progression (Fig 3). The first site of failure was the local area in 16 patients and distant sites in 27 patients. Eight patients showed recurrence in both the local and distant areas. Among 24 patients with locoregional failure, six patients showed out-of-field failure. The sites of out-of-field failure compared with the RT field are described in Table 3. For 35 patients with distant metastasis as the first site of failure, peritoneal seeding was the most common involvement site (n = 15), followed by the liver (n = 10), lymph node (n = 7), and lung (n = 3).

**Table 3:** Site of out-of-field failure and target volumes.

No.	Site of initial recurrence	CTV	Failure site
1	Portohepatis LN	No	Perihepatic space
2	Hepatic RM	Around RM	Around CHA area
3	Hepatic hilum	No	LN around hepatojejunostomy
4	Around SMA	No	Choledochoenterostomy site
5	Around IVC	No	Around pancreatic head
6	Hepatic hilum	No	Around celiac trunk

CTV, clinical target volume; LN, lymph node; RM, resection margin; CHA, common hepatic artery; SMA, superior mesenteric artery; IVC, inferior vena cava.

One patient experienced grade 3 neutropenia during CCRT and recovered with conservative management. During follow-up, two patients developed late grade 3 toxicities. One patient with duodenal bleeding recovered after an endoscopic intervention and one patient with duodenal obstruction received stent insertion.

## Discussion

Although surgical resection is the only curative treatment with long-term survival for patients with locoregional recurrence of BDC, most patients are not suitable as candidates for surgery due to unresectable local disease or poor expected survival over treatment-related morbidity. Because chemotherapy has been widely applied for initially unresectable or metastatic BDC, it has been used as the main treatment for locoregional recurrence of BDCs as well. One study on unresectable or metastatic biliary tract cancer showed that gemcitabine plus cisplatin resulted better survival outcomes compared with gemcitabine alone [13]; however, this result cannot be directly applied for recurrent EHBDC because the patient group was heterogeneous and included locally advanced or metastatic bile duct cancer, gallbladder cancer, and ampullary cancer [13]. In this study, RT for recurrent EHBDC showed favorable survival and local control, showing improved FFLP, PFS, and OS with higher RT dose, and improved PFS and OS with concurrent chemotherapy.

Locoregional recurrence of BDC may have a different prognosis compared with locally advanced or metastatic biliary cancer at the time of diagnosis. Although surgical resection for recurrent BDC could be limited, previous studies on surgical resection reported a median survival of 26 months with a 5-year survival rate of 29% [6, 7, 14]. Also, a multicenter retrospective study on the identification of prognostic factors for bile duct cancers treated with palliative chemotherapy reported that recurrent BDC had a significantly longer survival compared with locally advanced disease [15]. Collectively, there is a need for an effective local modality for locoregionally recurrent BDC because clinical outcomes after chemotherapy remain unsatisfactory and surgery is limited for patients with locoregionally recurrent BDC.

RT has been regarded as an option for the treatment of locally advanced bile duct cancer [16–20]. For unresectable BDC, local progression is a major cause of cancer death and local control is important to improve the survival outcomes [19–21]. Several studies reported that the efficacy of RT for unresectable EHBDC in terms of 2-year OS ranged from 13% to 41%, and an analysis using the Surveillance, Epidemiology, and End Results (SEER)-Medicare database reported that RT with chemotherapy showed improved survival [16–18, 22, 23]. Currently, CCRT with current fluoropyrimidine is recommended as one of the treatment options in clinical practice guidelines [24].

Although there is a paucity of reports on salvage RT for locoregional recurrence of biliary tract cancer after curative aimed surgery, retrospective studies with small patient populations have been published (Table 4) [8, 9, 12]. In these series, a significant proportion of patients received concurrent chemotherapy during RT (76%–100%) and 2-year locoregional progression-free survival and 2-year OS ranged from 34% to 54% and from 44% to 55%, respectively. In the current study, we analyzed 76 patients with recurrent cancer of only extrahepatic bile duct origin. The rates of 2-year FFLP and OS were 61% and 33%, respectively, and treatment-related toxicities were mild. Because the existing studies included a limited number of selective patients with varying characteristics, a direct comparison between the study results may not be appropriate (Table 4). However, the survival results seem superior compared with a clinical trial on chemotherapy for advanced biliary disease in which the median survival was less than one year [13]. Another notable result is that the 1-year FFLP (52%) and OS (47%) in the patients with RT alone seems not inferior to the results from previously reported studies on salvage RT or chemotherapy, although there exists lack of experience to derive the definite effectiveness of RT alone. Limitations on clinical experience on salvage RT still remain, and the result of the present study may thus be helpful in determining the optimal treatment modality for locoregional recurrence of EHBDC.

**Table 4:** Salvage radiation therapy for locoregional recurrence of extrahepatic bile duct cancer.

Study	Year	No.	DFI > 1-year	CCRT	Maintenance CT	2-yr LPFS	2-yr PFS	2-yr OS	Prognostic factor on OS
Kim et al. ^a^	2015	25	48%	76%	12%	44% (14)	15% (9)	55% (24)	CCRT, CA19-9
Kim et al.	2017	23	43%	78%	30%	54% (NA)	49% (15)	44% (18)	DFI, initial T-stage
Yu et al. ^a^	2017	42	37%	100%	29%	34% (15)	19% (10)	56% (41)	concurrent CT regimen, CA 19–9, initial T-stage
Current study	2021	76	49%	80%	32%	61% (33)^b^	25% (9)	33% (16)	CCRT, RT dose, DFI

Numbers in parenthesis are median values of the survival time (month).

^a^These studies included patients with recurrent biliary tract cancer originating from other than extrahepatic bile duct: n = 3 (ampulla of vater) and n = 9 (ampulla of vater, gallbladder, and intrahepatic bile duct).

^b^Freedom from locoregional progression was evaluated in this current study.

DFI, disease-free interval; CCRT, concurrent chemoradiation therapy; CT, chemotherapy; LPFS, locoregional progression-free survival; PFS, progression-free survival; OS, overall survival; CA 19–9, Carbohydrate antigen 19–9; NA, not assessed; RT, radiation therapy.

Patients with recurrent EHBDC after curative surgery could be a heterogeneous group with different prognosis [6, 25]. Theoretically, local salvage therapy would be more helpful for patients with a good prognosis compared with those who show early systemic progression after recurrence. Several factors were reported as possible prognostic factors for survival, including age, performance status, initial stage, RM status, tumor histology, DFI, surgery for recurrence, and the level of CA 19–9 [6, 8, 9, 12, 25]. In the present study, patients with DFI of more than 1-year showed a significantly better survival on univariate and multivariate analysis. DFI was reported as a significant prognostic factor for recurrent BDC in previous studies [6, 9, 25]. Although one study with a small number of patients showed contradictory results [9], early recurrence after initial surgery for biliary tract cancer has been generally reported to be related to poor prognosis [6, 25]. Moreover, a large retrospective study on patients with resected pancreatic cancer reported that DFI of 1-year was the optimal criteria for dividing between early and late recurrence, and that the early recurrence group showed poor 2-year post-recurrence survival than did the late recurrence group (6% vs. 22%, p < 0.001). Although there have been some debates on the differences between pancreatic cancer and EHBDC on prognosis and clinical behavior [26], DFI could be one of the surrogate markers for sub-dividing the patients and selecting the optimal treatment for recurrent pancreatobiliary diseases. Furthermore, DFI could be used for stratification of the patients who are potential candidates for RT with recurrent EHBDC, and optimization of RT scheme for these patients.

In pancreatobiliary cancer, a higher RT dose has been shown to be associated with improved LC and OS [19, 27, 28]. Crane et al. suggested that higher RT dose could be associated with improved LC in their retrospective analysis of patients with localized unresectable BDC who received RT with conventional techniques [18]. About a decade after this report, one study on RT dose escalation with precision RT technique using intensity-modulated RT or proton beam therapy showed that higher RT dose was significantly correlated with better LC and OS in patients with IHBDC [19]. In the present study, we found that an RT dose (BED) of 59 Gy or higher dose was a significant prognostic factor for LC, FFLP, and OS. Although there has been some controversy on the efficacy of dose-escalation for EHBDC [20], further clinical studies using modern RT techniques are needed to determine the optimal RT dose for locoregional recurrence of EHBDC. Also, high-precision RT techniques for lesions adjacent to critical organs may be helpful for further dose escalation with an acceptable rate of toxicity [20].

The optimal scheme of RT and chemotherapy for the treatment of locally advanced and recurrent BDC is yet to be established. In the current study, concurrent chemotherapy during RT was related to improved PFS and OS, and there was a trend for improved PFS with additional chemotherapy after RT. Considering the radiosensitizing effect of concurrent chemotherapy in gastrointestinal cancer and limited treatment-related toxicities in our study, CCRT could be considered a reasonable treatment modality for maximizing locoregional control. This suggestion is in line with the previous studies on the locoregional recurrence of BDC, which reported significant relationships between concurrent chemotherapy and survival [8, 9].

Although local progression is a common cause of death in patients with unresectable BDC, distant metastasis is a still major pattern of failure in resected BDC [21, 29]. In the present study, the patients experienced distant failure more frequently than local progression as the first site of failure. Our results suggest that additional chemotherapy may be beneficial for improving PFS related to both local and distant control after RT. The impact of RT as local therapy could be maximized with a more effective systemic control with novel chemotherapy regimens, such as nab-paclitaxel plus gemcitabine-cisplatin which showed improved response rate and better OS compared with historic controls with gemcitabine-cisplatin [30]. This regimen may prove to be successful in a manner similar to the advances for locally advanced pancreatic cancer with FOLFIRINOX combined with CCRT [31]. Further study is needed to establish the optimal treatment strategy for combining CCRT and sequential chemotherapy including induction or maintenance chemotherapy.

This study has some limitations including its retrospective nature and the limited number of patients with tumors at different sites. The heterogeneous use of chemotherapy in conjunction with RT is another obstacle in the interpretation of the results for evaluating the effectiveness of RT. The selection bias and low statistical power should be considered when interpreting the results of the current study. However, all patients were treated in a single institute by the same medical team that used a consistent treatment strategy. Moreover, to our best of knowledge, this study included the largest number of patients for reporting the treatment outcome of salvage RT for recurrent EHBDC. Therefore, the results of this study may be helpful for deciding the ideal treatment for recurrent EHBDC, with improvements in RT techniques and systemic therapy.

## Conclusions

RT was an effective treatment modality with acceptable treatment related toxicities for locoregional recurrence of EHBDC and a higher RT dose was associated with a better prognosis. Further study on RT dose escalation is warranted and further evaluations are needed to improve the systemic control of recurrent EHBDC, considering that the majority of the patients experience distant metastases.
